# Do malaria vector control measures impact disease-related behaviour and knowledge? Evidence from a large-scale larviciding intervention in Tanzania

**DOI:** 10.1186/1475-2875-12-422

**Published:** 2013-11-15

**Authors:** Mathieu Maheu-Giroux, Marcia C Castro

**Affiliations:** 1 Department of Global Health & Population, Harvard School of Public Health, 665 Huntington Avenue, Bldg I, Room 1113, Boston, MA 02115, USA

**Keywords:** Malaria, *Plasmodium falciparum*, Vector control, Larviciding, Behaviour change, Bayesian statistics, United Republic of Tanzania

## Abstract

**Background:**

Recent efforts of accelerated malaria control towards the long-term goal of elimination had significant impacts in reducing malaria transmission. While these efforts need to be sustained over time, a scenario of low transmission could bring about changes in individual disease risk perception, hindering adherence to protective measures, and affecting disease-related knowledge. The goal of this study was to investigate the potential impact of a successful malaria vector control intervention on bed net usage and malaria-related knowledge.

**Methods:**

Dar es Salaam’s Urban Malaria Control Program was launched in 2004 with the aim of developing a sustainable larviciding intervention. Larviciding was scaled-up using a stepped-wedge design. Cross-sectional and longitudinal data were collected using a randomized cluster sampling design (2004–2008). Prevalence ratios (PR) for the effect of the larviciding intervention on bed net usage (N = 64,537) and household heads’ knowledge of malaria symptoms and transmission (N = 11,254) were obtained from random effects regression models.

**Results:**

The probability that individuals targeted by larviciding had used a bed net was reduced by 5% as compared to those in non-intervention areas (PR = 0.95; 95% credible intervals (CrI): 0.94-0.97) and the magnitude of this effect increased with time. Larviciding also led to a decline in household heads’ knowledge of malaria symptoms (PR = 0.88; 95% CrI: 0.83-0.92) but no evidence of effect on knowledge of malaria transmission was found.

**Conclusion:**

Successful control interventions could bring about further challenges to sustaining gains in reducing malaria transmission if not accompanied by strategies to avoid changes in individual knowledge and behaviour. This study points to two major research gaps. First, there is an urgent need to gather more evidence on the extent to which countries that have achieved significant decline in malaria transmission are also observing changes in individual behaviour and knowledge. Second, multidisciplinary assessments that combine quantitative and qualitative data, utilizing theories of health behaviour and theories of knowledge, are needed to optimize efforts of national malaria control programmes, and ultimately contribute to sustained reduction in malaria transmission.

## Background

The last decade witnessed a rapid scale-up of effective malaria control interventions supported by the mobilization of important programmes and initiatives [1]. The increased coverage of packages of interventions of proven efficacy is believed to have led to important declines in malaria transmission and disease burden, particularly after 2005, in some areas of sub-Saharan Africa [2-4]. Globally, it is estimated that malaria incidence has declined by 17% and that malaria mortality rates have been reduced by 26% since 2000 [5]. The persistent shrinking of the malaria map and shift from moderate/high to low malaria endemicity in some countries has important consequences on population-level immunity [6], and raises questions for programme managers and policy-makers regarding sustainability of the achievements to avoid resurgence, as observed in the past [7], and to pursue malaria elimination [8,9]. In fact, out of the 99 malaria-endemic countries, 34 have now set or are realistically considering elimination targets [10].

The Global Malaria Eradication Program (1955–1969) taught us that maintaining momentum when malaria transmission is declining is of prime importance to programmatic success [11]. One of the cardinal requirements for moving beyond control to elimination is to sustain high rates of effective coverage of control measures within a low transmission environment [12]. Reducing malaria to low transmission levels, however, could negatively impact disease risk perception by local communities, policy makers, and international funders [13-15]. Few studies thoroughly investigated the impacts of malaria control on individual health behaviour and disease-related knowledge. Qualitative evidence suggests that bed net usage could decrease following a reduction in mosquito nuisance and malaria transmission [13,16,17]. Further, lack of experience with episodes of malaria illness and inaccurate home diagnosis have been suggested as contributing factors to delays in appropriate treatment-seeking behaviour [18,19].

This paper addresses the issue of potential behaviour change following successful malaria control efforts. Specifically, the potential impact of a vector control strategy on malaria-related behaviour and knowledge is assessed using data from the Urban Malaria Control Programme (UMCP) in Dar es Salaam (United Republic of Tanzania) [20]. This programme was chosen because after three years of larval control the odds of individuals living in areas treated with larvicide being infected with malaria were 21% lower than those who lived in untreated areas [21]. This study’s hypothesis is that as mosquito density and malaria transmission are reduced in Dar es Salaam, three changes could happen. First, as fewer infections are observed, people do not perceive malaria as a major risk for their health (or that of their family), and therefore the use of protective measures is relaxed. Although this change was not observed in a recent qualitative study in Zanzibar, it was stressed as a real possibility in low transmission areas [22]. Second, as people witness fewer episodes of malaria in their immediate social network, their ability to recognize symptoms of the disease is reduced. Third, as the perception of malaria as a major health threat decreases, overall knowledge about disease transmission is progressively reduced as well. However, given the fact that the UMCP larval control activities were done on a weekly basis, and considering that the population was aware of the work of larval control personnel, there is a chance that the link between mosquitoes and malaria is not compromised by reduced transmission. Thus, this paper examines the effects of the larval control strategy in Dar es Salaam on: i) reported bed net usage; ii) knowledge of malaria symptoms; and, iii) knowledge that mosquitoes transmit malaria.

## Methods

### Study site

Dar es Salaam is the largest city of the United Republic of Tanzania with an estimated population of 2.7 million in 2005 [23]. The smallest administrative units is the ten-cell unit (TCU), which is usually comprised of ten to 20 houses, but may contain as many as 100 [24]. Malaria transmission in Dar es Salaam is year-round [25] and incidence of malaria often peaks after the rainy seasons.

### Data collection

The UMCP was launched in 2004 with the goal of developing a sustainable community-based larviciding intervention. From 2004 to 2008, a total of six randomized cluster-sampled household surveys were conducted in the targeted area [21]. For the first survey round, ten TCUs per ward were randomly drawn and all households in the selected TCUs were eligible to participate. From the second survey round onwards, TCUs selected in the first round were followed up longitudinally, and cross-sectional data were collected from ten additional TCUs. Upon obtaining informed consent, the location of each household was georeferenced and a detailed questionnaire was administered. Information collected included: i) house characteristics; ii) head of household; iii) use of protective measures; and, iv) individual characteristics of household members. An asset index was constructed by performing a principal component analysis of the household’s possessions and used as a proxy of socio-economic status (SES). A total of 48,525 individuals contributed information to the study and 9,379 of these were interviewed more than once. Including follow-up data, the total sample size is 64,537 data points, of which 11,254 are from household heads.

The larviciding intervention was rolled-out sequentially: it started in March 2006 in three wards, scaled up to nine wards in May 2007, and to all intervention areas in April 2008. More details about the UMCP design and data collection can be found elsewhere [20,21,26].

### Statistical analyses

The three main outcomes of this study are: i) reported bed net usage the night before the survey (any type of bed net); ii) household head’s knowledge of at least five malaria symptoms; and, iii) household head’s knowledge that mosquitoes transmit malaria. The larviciding intervention was lagged by five weeks, as described by Maheu-Giroux and Castro [21].

Random effect models where used to take into account clustering of individuals at the household and TCU levels in the regression models (Model 1). As the larviciding intervention was not randomized [21,26], the possibility that ward characteristics are correlated with the intervention cannot be eliminated. Therefore, sensitivity of the results was assessed by including ward fixed effects in the statistical models (Model 2). Finally, the possibility that the changes in preventive behaviours and malaria knowledge were not constant through time after initiation of larviciding activities was examined (Model 3). Since the outcomes are not rare events, reporting odds ratios overstates the relative risk association. Model-adjusted prevalence ratios (PR) were therefore calculated directly from logistic regressions using marginal standardization [27,28]. A Bayesian framework was chosen because it offered the flexibility to consider fixed effects and cluster-level random effects, and straightforward computations of the prevalence ratios (PR) and their credible intervals (CrI).

Covariates included in the final multivariate models were selected based on careful consideration of the following issues: i) subject-matter knowledge about confounding; ii) variable exhibiting sufficient variation; and, iii) extent of potential measurement errors. Covariates included in the model when the outcome is bed net usage were: age, gender, use of insect repellent, use of sprays, use of coil, living in a house with window screens, SES quintiles, weekly rainfall lagged by two weeks (including a quadratic term), and having been surveyed in a previous survey round. Since all models included both follow-up and cross-sectional data, controls for follow-up individuals were added in order to account for any potential Hawthorne effect [29], or the fact that individuals interviewed multiple times adapt their response to questions based on what is expected to be correct. As for the models where the outcome is either knowledge of malaria symptoms or knowledge of malaria transmission, variables controlled for were: age, gender, having been surveyed in a previous survey round, and SES quintiles. Effect modification of the intervention by age, the household’s head gender, and SES (dichotomized as richer *vs* poorer than the median) was investigated using the model that provided the best fit as indicated by the deviance information criterion. Details on model specifications, prior distributions, model fitting and convergence, and sensitivity analyses can be found in Additional file 1.

### Ethical considerations

Ethical approval was granted by the Medical Research Coordination Committee of the National Institute for Medical Research, Ministry of Tanzania (Reference #NIMR/HQ/R.8a/Vol. IX/279&234), and by the Harvard School of Public Health Institutional Review Board (Protocol #20323-101). Upon informing the study participants on the goal, specific objectives, risk and benefits of the study, written informed consent was obtained. For children younger than 18 years of age, the parent or guardian provided signed informed consent on their behalf.

## Results

Characteristics of study participants, stratified by larviciding phase and intervention status, are presented in Table 1. Given the survey design, the proportion of individuals surveyed during the rainy season exhibited marked differences; a larger proportion of interviews for the larviciding areas of the first larviciding phase and of the non-intervention areas of the second larviciding phase were performed during the rainy season. The proportion of heads of household between 50 and 64 years of age increased with time (as a result of aging and the fact that older household heads enrolled with time), and SES and house-proofing conditions also exhibited increasing trends with time.

**Table 1:** Characteristics of study participants stratified by larviciding phase and intervention status

**Variables**	**Baseline**	**First phase**		**Second phase**		**Third phase**
	**Control**	**Control**	**Larviciding**	**Control**	**Larviciding**	**Larviciding**
**Individual-level characteristics (n)**	**26,338**	**13,818**	**3,096**	**4,749**	**7,366**	**9,170**
Male sex	35.2%	35.5%	36.6%	36.3%	38.2%	39.4%
Age						
Younger than 5 years of age	15.4%	13.3%	13.3%	13.2%	11.5%	10.0%
Between 5 and 14 years of age	27.6%	27.9%	29.1%	28.4%	29.6%	31.2%
Between 15 and 29 years of age	28.5%	29.5%	29.3%	28.2%	28.9%	29.1%
Between 30 and 44 years of age	16.4%	17.3%	16.1%	18.4%	18.8%	18.4%
Between 45 and 59 years of age	7.2%	7.2%	7.7%	7.6%	7.1%	7.3%
Aged 60 years or above	4.9%	4.8%	4.5%	4.1%	4.1%	4.0%
*Missing*	0.1%	0.1%	0%	0%	0%	0%
Reported use of mosquito repellent	1.3%	4.2%	3.7%	2.5%	3.1%	3.3%
Reported use of coil	5.7%	8.4%	5.9%	5.9%	7.3%	5.8%
Interviewed during the rainy season	41.1%	47.5%	51.4%	51.8%	30.9%	38.3%
Previously surveyed participant (follow-up)	16.9%	31.0%	30.4%	32.4%	31.3%	27.5%
**Head of household and house characteristics (N)**	**5,127**	**2,505**	**522**	**726**	**1,099**	**1,275**
Male sex	64.2%	71.6%	71.6%	70.8%	73.3%	74.7%
Age						
Younger than 30 years of age	8.2%	2.4%	2.9%	3.0%	1.7%	1.6%
Between 30 and 49 years of age	48.1%	47.3%	43.9%	47.2%	50.8%	48.9%
Between 50 and 64 years of age	31.1%	37.0%	42.3%	39.5%	36.6%	38.4%
Aged 65 years or above	11.7%	13.1%	10.9%	9.9%	10.5%	10.6%
*Missing*	0.9%	0.2%	0%	0.3%	0.5%	0.5%
Occupation of the household head						
Business/Government/Formal sector	59.3%	66.3%	65.7%	58.3%	69.5%	77.3%
Farmer/Fisherman	2.2%	1.2%	1.5%	1.1%	0.9%	0.6%
Informal sector	19.2%	20.0%	18.4%	25.3%	17.0%	12.0%
Retired/No job/Domestic	17.9%	11.5%	13.8%	13.8%	11.7%	9.2%
*Missing*	1.4%	1.0%	0.6%	1.5%	0.8%	0.9%
Socio-economic status						
Lowest quintile	31.9%	18.5%	21.5%	5.1%	9.1%	7.0%
Second quintile	27.6%	24.2%	16.5%	19.6%	16.3%	14.8%
Third quintile	13.9%	18.2%	19.2%	20.9%	15.0%	19.2%
Fourth quintile	11.6%	21.5%	20.5%	26.9%	31.2%	28.3%
Highest quintile	15.0%	17.5%	22.4%	27.5%	28.4%	30.7%
Education level of household head						
Illiterate	6.2%	6.9%	4.2%	4.0%	2.9%	1.2%
Primary	58.9%	43.5%	48.5%	37.5%	32.7%	35.0%
Secondary	29.2%	44.2%	39.1%	55.9%	59.4%	59.8%
Tertiary	3.6%	4.5%	7.1%	1.9%	4.3%	3.4%
Other	0.3%	0%	0.4%	0%	0.1%	0%
*Missing*	1.8%	0.9%	0.8%	0.7%	0.6%	0.6%
House has window screens	23.7%	24.2%	45.6%	22.2%	30.3%	39.7%
House has whole ceiling	25.1%	29.5%	36.0%	44.5%	41.7%	34.8%

**Figure 1:**
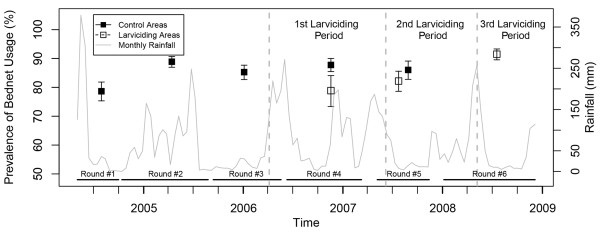
**Prevalence of bed net usage stratified by survey round and larviciding status.** Confidence intervals are based on 9,999 bootstrap replicates at the TCU levels. (The time frame of larviciding phases and survey rounds do not overlap perfectly. Thus, due to small sample size and the geographically limited extent of data collection (only one ward), results for 697 data points in the larviciding area in survey round 3, and 744 data points in control area in survey round 6 are not shown).

**Figure 2:**
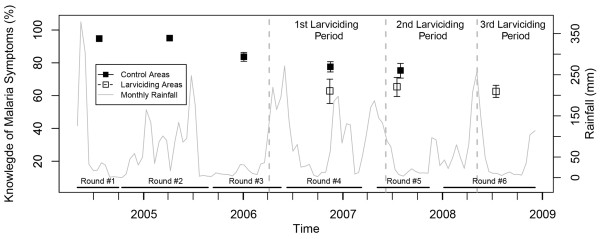
**Proportion of household heads knowing at least five symptoms of malaria, stratified by survey round and larviciding status.** Confidence intervals are based on 9,999 bootstrap replicates at the TCU levels. (Prevalence estimates based on small sample size and geographically limited extent of data collection are not represented).

**Figure 3:**
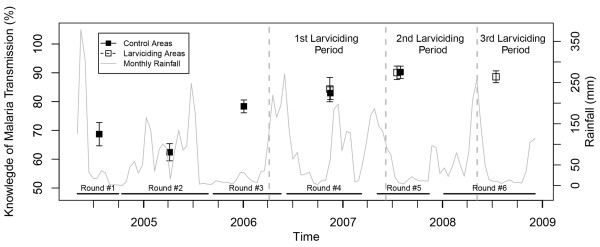
**Proportion of household heads that know that mosquitoes transmit malaria, stratified by survey round and larviciding status.** Confidence intervals are based on 9,999 bootstrap replicates at the TCU levels. (Prevalence estimates based on small sample size and geographically limited extent of data collection are not represented).

Reported use of bed net increased steadily in the non-intervention areas from 78.7% in mid-2004 to 86.0% in 2007, but exhibited yearly variation related to precipitation (Figure 1), and was lower in larviciding areas as compared to non-intervention ones. With regard to knowledge of malaria symptoms by the household head, a continuous decline was observed throughout the study period in non-intervention wards from 94.8 to 75.3% (Figure 2), and in larviciding wards from 62.9 to 62.6%. The proportion of household heads with knowledge that mosquitoes transmit malaria rose steadily during the study period in the non-intervention group from 68.7 to 90.2% (Figure 3), and non-intervention and larviciding areas did not appear to differ much.

Univariate regression models suggested that the probability of using a bed net the night before the survey for individuals residing in larviciding areas was reduced by 6% (95% CrI: 4-7%) as compared to individuals living in non-intervention areas (Table 2). This result was not affected when adjusting for additional covariates and when including fixed effects at the ward level. When examining if the intervention only had an immediate effect or one that changes with time, the decline in bed net usage observed in the larviciding wards was found to be accentuating with time (Table 2) so that, after three years of larviciding, the probability of using a net for individuals living in the intervention wards was reduced by 10% (PR = 0.90, 95% CrI: 0.84-0.95) as compared to individuals in non-intervention wards.

**Table 2:** Effect size estimates of the larviciding intervention on reported bed net usage the night before the survey

**Outcome: Bed net usage (N = 64,537** **)**	**Model 1**		**Model 2**		**Model 3**	
	PR*	**95% CrI†**	PR*	**95% CrI†**	PR*	**95% CrI†**
**Univariate**						
Larviciding intervention	**0.94**	**(0.93-0.96)**	**0.94**	**(0.93-0.96)**	**0.95**	**(0.93-0.96)**
Time since initiation of larviciding (years)	-	-	-	-	**0.98**	**(0.96-0.99)**
**Multivariable‡**						
Larviciding intervention	**0.96**	**(0.94-0.97)**	**0.95**	**(0.94-0.97)**	**0.96**	**(0.94-0.97)**
Time since initiation of larviciding (years)	-	-	-	-	**0.98**	**(0.97-0.99)**
*Trend for time (AR1§)*	*Yes*		*Yes*		*Yes*	
*Random effects (Household and TCU)*	*Yes*		*Yes*		*Yes*	
*Fixed effects at ward level*			*Yes*		*Yes*	

Statistically significant results are bolded.

To account for the fact that the coefficients of the ward fixed effects exhibited slow convergence, the number of iterations used for inference was doubled to 120,000 for Models (2) and (3).

*PR: Prevalence ratio.

†CrI: Credible interval.

§AR1: First-order autoregressive.

‡Control variables include: age, gender, dummy for being a follow-up observation, use of insect repellent, use of sprays, use of coil, living in a house with window screens, socio-economic status, and weekly rainfall lagged by two weeks (with quadratic term).

The impact of the larviciding intervention on knowledge of malaria symptoms was also shown to be statistically significant (Table 3). Here, adding fixed effects at the ward level slightly changed the PR for the intervention from 0.91 (95% CrI: 0.87-0.95) to 0.88 (95% CrI: 0.83-0.92). The PR were unaffected when adjusting for potential confounders. Further, time since initiation of larviciding activities had no effect on knowledge of malaria symptoms.

**Table 3:** Effect size estimates of the larviciding intervention on knowledge of at least five malaria symptoms

**Outcome: Symptoms knowledge (N = 11,254** **)**	**Model 1**		**Model 2**		**Model 3**	
	PR*	**95% CrI†**	PR*	**95% CrI†**	PR*	**95% CrI†**
**Univariate**						
Larviciding intervention	**0.91**	**(0.87-0.95)**	**0.88**	**(0.83-0.92)**	**0.87**	**(0.82-0.92)**
Time since initiation of larviciding (years)	-	-	-	-	1.03	(0.99-1.07)
**Multivariable‡**						
Larviciding intervention	**0.91**	**(0.87-0.95)**	**0.88**	**(0.83-0.92)**	**0.87**	**(0.82-0.92)**
Time since initiation of larviciding (years)	-	-	-	-	1.01	(0.98-1.05)
*Trend for time (AR1§)*	*Yes*		*Yes*		*Yes*	
*Random effects (TCU)*	*Yes*		*Yes*		*Yes*	
*Fixed effects at ward level*			*Yes*		*Yes*	

Statistically significant results are bolded.

*PR: Prevalence ratio.

†CrI: Credible interval.

§AR1: First-order autoregressive.

‡Control variables include: age, gender, dummy for being a follow-up observation, and socio-economic status.

No evidence supporting a change in knowledge of malaria transmission as a result of the larviciding intervention was found (Table 4). Results were not affected by adding fixed effects at the ward levels or by adjusting for potential confounders. When allowing for a change of the effect of the intervention with time, the results suggested that household heads living in larviciding areas were less likely to recognize mosquitoes as vector of malaria as time since initiation of larviciding activities increased. Indeed, the model predicts that three years after initiation of the larval control intervention, the probability that household heads residing in larviciding areas recognized mosquitoes as vector of malaria was reduced by 10% (PR = 0.90; 95% CrI: 0.75-1.04) as compared to those living in non-intervention areas. This result did not reach statistical significance, however.

**Table 4:** Effect size estimates of the larviciding intervention on knowledge of malaria transmission

**Outcome: Knowledge of malaria transmission (N = 11,254)**	**Model 1**		**Model 2**		**Model 3**	
	PR*	**95% CrI†**	PR*	**95% CrI†**	PR*	**95% CrI†**
**Univariate**						
Larviciding intervention	1.01	(0.96-1.05)	1.00	(0.95-1.05)	1.01	(0.95-1.06)
Time since initiation of larviciding (years)	-	-	-	-	0.97	(0.92-1.02)
**Multivariable‡**						
Larviciding intervention	1.01	(0.96-1.05)	1.00	(0.95-1.05)	1.02	(0.97-1.07)
Time since initiation of larviciding (years)	-	-	-	-	0.96	(0.92-1.01)
*Trend for time (AR1§)*	*Yes*		*Yes*		*Yes*	
*Random effects (TCU)*	*Yes*		*Yes*		*Yes*	
*Fixed effects at ward level*			*Yes*		*Yes*	

Statistically significant results are bolded.

*PR: Prevalence ratio.

†CrI: Credible interval.

§AR1: First-order autoregressive.

‡Control variables include: age, gender, dummy for being a follow-up observation, and socio-economic status.

**Figure 4:**
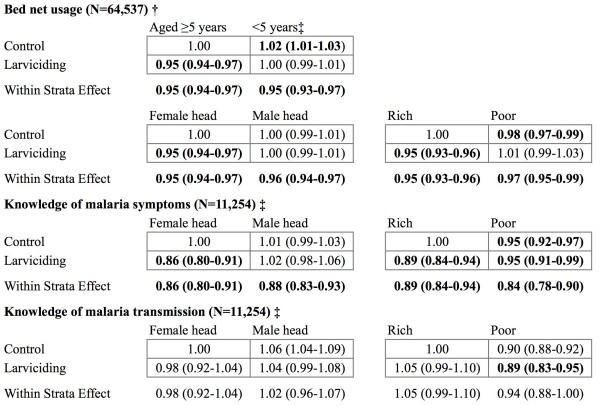
**Effect modification of the larviciding intervention by age, gender, and socio-economic status on bed net usage, knowledge of malaria symptoms, and knowledge of malaria transmission.** Statistically significant results are bolded. To account for the fact that the coefficients of the ward fixed effects exhibited slow convergence for the ‘Bed net usage’ models, the number of iterations used for inference was doubled to 120,000. † Models for the bed net usage outcome are adjusted for: age, gender, dummy for being a follow-up observation, use of insect repellent, use of sprays, use of coil, living in a house with window screens, socio-economic status, and weekly rainfall lagged by two weeks (with quadratic term). Models also include: a semiparametric time trend, random effects at household and TCU levels, and fixed effects at the ward level (as in Model 2). ‡ Models for the knowledge of malaria symptoms and malaria transmission outcomes are adjusted for: age, gender, dummy for being a follow-up observation, and socio-economic status. Models also include: a semiparametric time trend, random effects at TCU level, and fixed effects at the ward level (as in Model 2).

Finally, neither being under five years old, living in a household headed by a male, nor being below the median SES was found to be modifying the effect of the larviciding intervention on reported bed net usage (Figure 4). For both the knowledge of malaria symptoms and malaria transmission outcomes, the product term between the larviciding intervention and gender of the household head was not statistically significant, indicating that this variable is not an effect modifier. Being below the median SES asset-based index, however, significantly modified the effect of the larviciding intervention on malaria knowledge. In fact, the PR for the larviciding intervention for heads of household above the median SES was 0.89 (95% CrI: 0.84-0.94) as compared 0.84 (95% CrI: 0.78-0.90) for those living below the median SES. Even though the product term between SES and the larviciding intervention reached statistical significance for knowledge of malaria transmission, the CrI of the SES stratum-specific PR crossed the null.

## Discussion

These results showed that individuals targeted by the larviciding intervention in Dar es Salaam were significantly less likely to have used a bed net the night before the survey. The magnitude of this effect increased with time such that, three years after the initiation of larviciding activities, individuals in intervention areas were 10% less likely to use their bed net as compared to individuals living in non-intervention areas. There was also a decline in household heads’ knowledge of malaria symptoms and this effect was more pronounced for individuals of low SES. No differences between larviciding and non-intervention areas, with respect to knowledge of malaria transmission, were found.

With regard to bed nets, several studies have suggested that their use is a function of night-time temperature, perceived malaria risk and density of nuisance biting insects [30-32]. Thus, the significant reduction in the probability of using a bed net in UMCP intervention areas could result from two factors. First, the UMCP made a programmatic decision to control larval stages of nuisance biting insects such as *Culex quinquefasciatus* (a mosquito involved in the transmission of lymphatic filariasis, but not malaria), as an effort to gain community support. A significant reduction in nuisance biting rates could deter individuals from using bed nets if personal protection against mosquito bites is not perceived as being necessary anymore. Nevertheless, data from the first phase of the UMCP intervention suggest that routine larviciding was not successful in suppressing nuisance biting, and culicine mosquitoes were still responsible for more than 100 bites per exposed person per night in the intervention wards [20]. The impact of controlling nuisance biting insects will be context specific, however, depending on the relative abundance of different species of mosquitoes. Second, the reduction in the prevalence of malaria infection from 20.8% in 2004 to 1.7% in 2008 following larval control [21,26] can potentially change the individual perception of malaria risk. In this case, the disease may not be perceived as a threat to health anymore, leading to varied behaviour changes, including reduced adoption of personal protective measures, such as bed net use. The reported results tend to support this hypothesis.

Despite the significant reduction in the probability of using a bed net following the larviciding intervention, the proportion of individuals using a net in non-intervention areas increased throughout the study period. In October 2004, shortly after the initiation of this study, the Tanzania National Voucher Scheme was launched*.* The aim of this programme was to provide every pregnant woman with a printed voucher valued at TZS2,750 (USD2.75 in 2004) to purchase a discounted-price bed net [33]. In October 2006, a second voucher was introduced targeting mothers and caretakers of infants aged nine months at the time of measles vaccination [34] and, in January 2007, the value of the voucher was increased to TZS3,250 [35]. The subsequent introduction and improvements of these financial incentives could thus have resulted in higher bed net ownership and usage.

A decline in the knowledge of malaria symptoms, particularly in areas under the UMCP larval control intervention, is also worrisome. Caregivers’ inability to recognize malaria symptoms has been cited as an impeding factor for early treatment of severe malaria in Tanzania [19]. With lower transmission intensities, population-level immunity is expected to decrease and the clinical spectrum of severe malaria may change with cerebral malaria accounting for a higher proportion of cases [6]. Therefore, early and proper recognition of symptoms is crucial to reduce malaria morbidity and mortality [36]. Of particular concern is the finding that SES is modifying the relationship between larviciding and knowledge of malaria symptoms. Given that out-of-pocket expenditure for malaria treatment usually consumes a larger proportion of low SES households’ budget [37], inappropriate or delayed treatment could potentially be exacerbated in these disadvantaged households by their inability to recognize malaria symptoms.

If knowledge is formed based on experience, one could hypothesize that as malaria transmission goes down, and fewer cases are observed, personal experience with malaria episodes also reduces, and thus the ability of individuals to properly identify disease symptoms may be compromised. That would be maximized if malaria was not perceived as a major threat. While intuitively it is reasonable to assume that these changes would increase over time (assuming that transmission remains fairly low or declines even further), this study’s results do not support that. In addition, the available data do not allow assessing the mechanisms through which knowledge of malaria symptoms is changed.

Regarding knowledge that mosquitoes transmit malaria, there is no evidence of changes following the UMCP larval control. Two factors could explain this result. First, community sensitization and participation are a central component of an integrated vector management strategy as endorsed by the World Health Organization [38]. In Dar es Salaam, each TCU has a leader and the UMCP worked closely with them to foster support for the larviciding activity, and to guarantee unrestricted access to breeding habitats, many located on private properties. Therefore, the population living in the UMCP area was aware of the presence and the purpose of larval control teams. Second, larval control personnel conducted their work wearing a UMCP T-shirt, displaying the name of the project and the life cycle of the mosquito. Thus, the weekly presence of the larval control teams may have acted as a regular reminder of the importance of mosquitoes for malaria transmission. These two factors could potentially overcome the expected decline in knowledge in scenarios of low malaria transmission.

The strengths of this study include its large geographic and temporal extents, availability of reliable baseline information, control of many potential confounders, reporting of effect size estimates on the risk ratio scale, a large sample size, and detailed use of robustness checks and sensitivity analyses. The study has some limitations. First, the order of the rollout of the intervention was not randomly allocated. If ward-level characteristics are correlated with the intervention, the reported effect size estimates could be biased. Nevertheless, including fixed effects at the ward level, which control for ward-level time-invariant confounders, did not affect the reported effect size estimates. Second, information on knowledge of malaria symptoms and transmission was only collected from household heads. Intra-household decisions about health expenditure and treatment-seeking behaviour follow a complex process that involves trade-offs and bargaining among household members. This paper’s inferences are thus based on the assumption that the household head’s level of malaria knowledge is representative of that of other household members involved in this decision making process. The fact that gender was not found to be an effect modifier tends to support this assumption.

This study’s findings need to be discussed in light of the current efforts of intensified malaria control with the goal of eradication. In countries considering elimination, and in areas where transmission has been reduced to very low levels for a few years, acquired immunity is low and thus sustaining gains of malaria control becomes crucial to prevent outbreaks and resurgence of the disease [11], such as that occurred in Sri Lanka during the late 1960s [7]. If knowledge and behaviour change follows successful interventions that reduce malaria transmission to low levels, then sustainability of control efforts and gains may be at risk. A potential strategy to address these issues, currently largely neglected by national malaria control programmes, is the implementation of a comprehensive behavioural change communication process, which addresses gaps in knowledge and problems in disease risk perception.

## Conclusions

This study points to two major research gaps. First, there is an urgent need to conduct more studies, similar to this one, to assess the extent to which countries that have achieved significant decline in malaria transmission are also observing changes in individual behaviour and knowledge. Second, multidisciplinary assessments that combine quantitative and qualitative data, utilizing theories of health behaviour and theories of knowledge, are needed to inform and optimize efforts of national malaria control programmes, and ultimately contribute to sustained reductions in malaria transmission.

## Competing interests

The authors have declared that they have no competing interests.

## Authors’ contributions

MCC developed the original research idea of the paper, designed the UMCP household survey and supervised data collection with inputs from UMCP collaborators, advised on data analysis and interpretation, and edited the manuscript. MMG performed the data analyses, interpreted the results, and wrote the manuscript. Both authors read and approved the final manuscript.

## Supplementary Material

Additional file 1 Details on model specifications, prior distributions used, model fitting and convergence diagnostics, and performed sensitivity analyses. Click here for file
