# Supplementary material for: Do malaria vector control measures impact disease-related behaviour and knowledge? Evidence from a large-scale larviciding intervention in Tanzania
Source: Malar J. 2013 Nov 15;12:422. doi: 10.1186/1475-2875-12-422 (PMC3835455; doi:10.1186/1475-2875-12-422)
Supplement: Additional file 1 — Details on model specifications, prior distributions used, model fitting and convergence diagnostics, and performed sensitivity analyses. [file 1475-2875-12-422-S1.doc]

**Additional File 1:**

**Do Malaria Vector Control Measures Impact Disease-Related Behaviours and Knowledge? Evidence from a Large-Scale Larviciding Intervention in Tanzania**

*Mathieu Maheu-Giroux^1^ & Marcia C. Castro^1,*^*

^1^ Department of Global Health & Population, Harvard School of Public Health, Boston MA.

** Corresponding author*

In this supplementary appendix, we provide detailed information on the statistical analyses we performed. We have organized this supplement in four sections. First we describe in greater details the three model specifications used in the manuscript. Second, the different prior distributions for the models’ parameters and hyperparameters are defined. Third, we described how the models were fitted and the type of convergence diagnostic performed. Lastly, we describe some of the sensitivity analyses we performed to assess the robustness of our findings.

**Model specifications**

We assumed that our binary outcomes followed a Bernoulli distribution, *Y_i_* ~ *Bernoulli(p_i_)*, where *p_i_* is the probability of an individual having the outcome, which is itself a function of covariates modelled with a *logit* link. We present three models with increasing levels of complexity, as described below.

Model 1: TCU and household random effects model

$$\text{logit}(p_{itjk})=\text{ }\alpha+\beta(\text{Intervention}_{\text{it}})+\delta X_{it}+f(\text{Time})+\mu_{\text{j}}+\upsilon_{k}+\varepsilon_{itjk}$$

$$\mu_{j}\sim N(0,\sigma_{\mu}^{2}),\text{ }\upsilon_{\text{k}}\sim N(0,\sigma_{k}^{2})\text{, and }\varepsilon_{\text{itjk}}\sim N(0,\sigma^{2})$$

where *p_itjk_* is the probability of individual *i* at time *t* living in TCU *j* and, if applicable, household *k*, to have the outcome of interest (i.e., used a bednet, know malaria symptoms, and know how malaria is transmitted); *β* is the coefficient of the larviciding intervention; *δ* is a vector of coefficients for control variables in *X*; *μ_j_* is a TCU-level random effect; and *υ_k_* is an household random effect. Note that the household level random intercept is included only when the outcome is bednet usage (individual level variable) as information on knowledge of malaria symptoms and transmission was recorded for household heads only. Finally, the time trend was accounted for with *f(.)*, a semi-parametric smooth function where a spline penalty follows a first order autoregressive process [1].

Model 2: TCU and household random effects model with ward fixed effects

$$\text{logit}(p_{itjk})=\text{ }\alpha+\beta(\text{Intervention}_{\text{it}})+\delta X_{it}+\omega\text{Ward}_{it}+f(\text{Time})+\mu_{\text{j}}+\upsilon_{k}+\varepsilon_{itjk}$$

where *p_itjk_* is the probability of individual *i* at time *t* living in TCU *j* and, if applicable, household *k* to have the outcome of interest; ω is a vector of coefficients for the ward fixed effects; and *β, δ_d_*, *f(.)*, *μ_j_*, and *υ_k_* are similar to those described in Model 1.

Model 3: TCU and household random effects model with ward fixed effects and allowing for change in slope as a function of time since initiation of intervention

$$\begin{matrix} \text{logit}(p_{itjk})=\text{ }\alpha+\beta(\text{Intervention}_{\text{it}})+\gamma(\text{Time } \text{Since Intervention}_{\text{it}})+ \\ \delta X_{it}+\omega\text{Ward}_{it}+f(\text{Time})+\mu_{\text{j}}+\upsilon_{k}+\varepsilon_{itjk} \end{matrix}$$

where *p_itjk_* is the probability of individual *i* at time *t* living in TCU *j* and, if applicable, household *k* to have the outcome of interest; *β* is the coefficient for the level shift in the outcome due to the intervention; γ is the coefficient for the change in slope as a function of time (in years) since the initiation of the larviciding activities; and ω*, δ_d_*, *f(.)*, *μ_j_*, and *υ_k_* are similar to those described in Models 1 and 2.

**Prior distributions**

Priors for the regression parameters were assumed to have non-informative Gaussian (mean = 0, precision= 0.001) distributions. Priors for the standard deviations of the random effects at the TCU and household levels were assumed to follow non-informative Uniform (0, 100) distributions. The hyperparameters for the first order autoregressive semi-parametric smooth were given a Gamma (shape = 1, scale = 1e-5) prior for the precision parameter, as proposed by Natário and Knorr-Held [2], and a Gaussian (mean = 0, precision = 0.40) prior for the first lag correlation parameter, which was defined on the logit scale. In order to improve mixing of the MCMC chains and faster convergence, hierarchical centering and parameter expansion were used [3, 4].

**Model fitting**

The Bayesian models were fitted using Markov Chain Monte Carlo (MCMC) simulations. All analyses were performed using the R statistical software [5]. Estimation of the marginal posterior distribution of the parameters of interest was performed using JAGS [6, 7]. An adaptive phase of 5,000 iterations and a minimum of 65,000 iterations from the Metropolis-Hasting algorithm were used for inferences (5,000 iterations used as burn-in). Convergence and stationarity were assessed through visual inspection of trace plots, the Raftery-Lewis statistic [8], and the Heidelberger and Welch's diagnostic [9]. The ‘rjags’ and ‘CODA’ libraries were used as an interface to run JAGS directly from R [10] and perform convergence diagnosis [11], respectively. Observations with missing data for age (n=44) were retained in the analysis using the missing indicator method [12].

**Sensitivity analyses**

The robustness of our results to model specification was also investigated. Specifically, we examined the sensitivity of the choice of penalty type for the time trend (1^st^ order autoregressive versus 1^st^ and 2^nd^ order random walks), different covariates adjustments (SES versus educational level and occupation), potential spillover effects (contamination of our intervention wards from non-intervention areas), and presence of spatially structured effects using Conditionally Auto-Regressive models [13]. Results from these sensitivity analyses demonstrated that our reported effect size estimates were robust to our modelling assumptions and only the three main models described above will be reported in this paper.

**References**

1. Congdon P: *Bayesian statistical modelling.* 2nd edn. Chichester, England ; Hoboken, NJ: John Wiley & Sons; 2006.

2. Natario I, Knorr-Held L: **Non-parametric ecological regression and spatial variation.** *Biometrical Journal* 2003, **45:**670-688.

3. Gelfand A, Sahu S, Carlin B: **Efficient parametrizations for normal linear mixed models.** *Biometrika* 1995, **82:**479-488.

4. Liu C, Rubin D, Wu Y: **Parameter expansion to accelerate EM: The PX-EM algrotihm.** *Biometrika* 1998, **85:**755-770.

5. R Development Core Team: **R: A Language and Environment for Statistical Computing.** Vienna, Austria: R Foundation for Statistical Computing; 2012.

6. Plummer M: **JAGS: A Program for Analysis of Bayesian Graphical Models Using Gibbs Sampling.** In *3rd International Workshopon Distributed Statistical Computing; Vienna, Austria*. Edited by Hornik K, Leisch F, Zeilei A. 2003: 1-10.

7. Plummer M: **JAGS Version 3.3.0 user manual.** pp. 412012:41.

8. Raftery A, Lewis S: **One long run with diagnostics: implementation strategies for Markov Chain Monte Carlo.** *Statistical Science* 1992, **7:**493-497.

9. Heidelberger P, Welch P: **Simulation run length control in the presence of an initial transient.** *Operations Research* 1983, **31:**1109-1144.

10. Plummer M: **rjags: Bayesian graphical models using MCMC. R package.**, Version 3-7 edition2012.

11. Plummer M, Best N, Cowles K, Vines K: **CODA: Convergence Diagnosis and Output Analysis for MCMC.** *R News* 2006, **6:**7-11.

12. Miettinen OS: *Theoretical epidemiology : principles of occurrence research in medicine.* New York: Wiley; 1985.

13. Besag J: **Spatial interaction and statistical analysis of lattice systems.** *Journal of the Royal Statistical Society Series B-Methodological* 1974, **36:**192-236.
